# Predictors for treatment outcomes after corneal crosslinking for keratoconus: a validation study

**DOI:** 10.1007/s10792-016-0262-z

**Published:** 2016-05-24

**Authors:** Daniel A. Godefrooij, Kim Boom, Nienke Soeters, Saskia M. Imhof, Robert P. L. Wisse

**Affiliations:** Utrecht Cornea Research Group, Department of Ophthalmology, University Medical Center Utrecht, Office E03.136, PO Box 85500, 3508 GA Utrecht, The Netherlands

**Keywords:** Keratoconus, Crosslinking, Prognosis, Multivariable analysis

## Abstract

Previous research suggested that baseline corrected distance visual acuity (CDVA) and maximum keratometry (Kmax) are the predictors for effectiveness of corneal crosslinking (CXL) for keratoconus. The aim of this study was to validate the previously determined predictors in a new treatment cohort. A prospective cohort of 112 eyes in 90 consecutive patients was used to validate the results of 102 eyes in 79 patients from our previous prospective cohort. All patients were treated using epithelium-off corneal CXL in a tertiary hospital setting. Primary outcomes were changes in CDVA (LogMAR) and Kmax between baseline and 1-year post-treatment. Predictive factors for both outcomes were determined using univariable and multivariable analyses. Lower pretreatment CDVA was found to be the sole independent factor predicting an improvement in CDVA 1 year after CXL (β coefficient: −0.476, *P* < 0.01). Kmax flattening is more likely to take place in eyes with preoperative central cones (β coefficient: 0.655, *P* < 0.01). These results are consistent with our initial research and indicate high reproducibility in the new cohort. The previously postulated prediction model for postoperative CDVA showed limited predictive value in the validation cohort (*R* ^2^ = 0.15). The clinical implication of these results is that patients with lower pretreatment visual acuity are more likely to benefit from CXL (with respect to visual acuity), and patients with more central cones will benefit more in terms of cone flattening. Furthermore, those results can be used to guide customization of the crosslinking treatment.

## Introduction

Keratoconus is a progressive disease in which protrusion of the cornea causes visual impairment through the formation of irregular astigmatism [1, 2]. The typical age of onset for keratoconus is early adulthood, and the disease is likely multifactorial in origin [3]. Genetic factors, environmental factors, positive family history, atopic constitution, contact lens use, and eye-rubbing have all been associated with keratoconus [4–9]. To halt disease progression, corneal crosslinking (CXL) has shown promising results in patients with keratoconus [10–12]. However, continued disease progression and a further decrease in visual acuity have been reported following CXL [13].

Many factors that might be related to the efficacy of CXL have been studied previously. For example, age is associated with changes in visual acuity, as pediatric patients show better improvement than older patients in terms of corrected distance visual acuity (CDVA) following CXL [14, 15]. A Snellen CDVA value worse than 20/40 is also correlated with an improvement in visual acuity following CXL [16, 17]. With respect to flattening of maximum keratometry (Kmax) following CXL, higher pretreatment Kmax (≥54 D), a more central cone, and central cornea thickness ≥450 µm have all been reported as predictive factors [16–21]. The majority of these associations were established using univariable analyses, although CDVA is known to be influenced by many interrelated factors [22].

Predictors of CXL efficacy have previously been studied by our group in a consecutive treatment cohort; specifically, differences in CDVA and Kmax 1 year after CXL were assessed using a multivariable model [23]. Interestingly, baseline CDVA was the only independent factor for predicting change in CDVA 1 year after CXL, and cone eccentricity was the only independent factor associated with change in Kmax following CXL. Moreover, a reliable model for predicting post-CXL changes in CDVA was constructed (*R* ^2^ = 0.45). In order to determine the reliability and generalizability of this model, external validation of these findings is essential.

Predictors are often not generalizable to patients outside the study population. Our primary purpose was to validate the reproducibility of previously determined predictors in a new treatment cohort. Only after validation such results should be implemented in clinical practice. Our secondary purpose was to validate the previously published model for the prediction of visual outcomes for individual patients following CXL [23].

## Methods

### Dataset and study design

Our current cohort included patients who were treated with epithelium-off CXL for progressive keratoconus in our institution from January 1, 2012 to October 31, 2013. Here, we refer to this cohort as the validation cohort. The study design, inclusion and exclusion criteria, data collection, and surgical procedure were adapted from the initial treatment cohort, which included patients who were treated from January 1, 2010 through December 31, 2011 [23]. The inclusion criteria included a prior Kmax progression of ≥1.0 D within 6–12 months and thinnest corneal pachymetry ≥400 µm. Patients with corneal scarring or infection, pregnant patients, and lactating patients were excluded. The following primary outcomes were examined: (1) change in CDVA (logMAR CDVA) between baseline and 1-year post-CXL, and (2) change in Kmax between baseline and the 1-year post-CXL. This study was approved by the Ethics Review Board of the University Medical Center Utrecht and was performed in accordance with local laws, the European guidelines of Good Clinical Practice, and the tenets of the Declaration of Helsinki.

### Surgical procedure

After the corneal epithelium was removed, crosslinking was performed in accordance with the Dresden protocol, using UV radiation with a perpendicular emission plane (370 nm at 3 mW/cm^2^, UV-X, Peschke Meditrade GmbH, Waldshut-Tiengen, Germany) as described previously [22–25].

### Data collection

Standard measurements were obtained at all follow-up visits and included uncorrected distance visual acuity (UDVA), CDVA, manifest refraction, Scheimpflug corneal tomography (Pentacam HR type 70900, Oculus GmbH, Wetzlar, Germany), and slit lamp evaluation. Parameters were measured prior to treatment and at regular follow-up visits (1, 3, 6, 12, and 18 months of post-treatment). Patient-related factors, including family history, atopic constitution, and smoking history, were collected from the patient charts and supplemented using standardized forms completed by phone or e-mail in case they were not noted in the patient charts. Family history was considered positive if a first-degree or second-degree relative had been diagnosed with keratoconus. Patients with asthma, eczema, hay fever, or anti-allergy medication were marked as positive for atopic constitution. Patients who were current smokers or previous smokers were marked as smokers, and the number of pack-years was noted.

### Statistical analysis

Progression of keratoconus 1 year after CXL treatment was defined as an increase in Kmax ≥1 D. The paired Student’s *t* test was used to analyze the differences in logMAR CDVA and Kmax between baseline and the 12-month follow-up visit. Five patients missed the 12-month follow-up visit, but they did attend the 6- and 18-month visits; for these patients, simple longitudinal imputation was used to estimate their CDVA and Kmax values at 12 months [26].

In this validation cohort, univariable analysis was performed in order to identify factors associated with the primary outcome parameters (i.e., change in CDVA and Kmax). All factors with *P* ≤ 0.20 from the univariable analysis were entered into a multivariable linear regression analysis to identify independent predictive factors. This method is consistent with the statistical method used to analyze the initial treatment cohort. The analysis was performed using generalized estimating equations, with correction for patients in which both eyes were included in the study.

The prediction model, which was postulated based on the initial treatment cohort, was sequentially validated; pretreatment logMAR-transformed visual acuity measurements of the validation cohort were entered in the model. The predicted and observed differences in logMAR CDVA values were compared using linear regression and presented in a calibration plot. Discrimination was summarized using *R* ^2^ to quantify the model’s performance. A new prediction model based on the validation cohort was produced by stepwise, backward removal of the least significant factors derived from the multivariable analysis. To validate the performance of the refined prediction model, both calibration and discrimination were tested. A calibration plot and additional *R* ^2^ of the observed and predicted values were obtained. Data were collected and analyzed using SPSS 21.0 (IBM, Armonk, NY).

## Results

### Dataset characteristics

The validation cohort consisted of 112 eyes from 90 patients who were treated using CXL within the study period. Ten eyes were lost to follow-up due to the patients moving abroad (*n* = 2) or unknown reasons (*n* = 8). These patients did not differ from the remaining study sample with respect to their baseline characteristics. Patient-related factors were unknown in five patients. The baseline characteristics of the initial and validation cohorts were similar, and are summarized in Table 1.

**Table 1:** Baseline characteristics of the initial cohort of 102 eyes of 79 and validation cohort of 112 eyes of 90 keratoconus patients

	Initial cohort (102 eyes in 79 patients)			Validation cohort (112 eyes in 90 patients)		
	*N*	SD/%	Missing	*N*	SD/%	Missing
Age (years), mean	23	±8	0	23	±8	0
Male, *N*	56	71	0	59	66	0
Right eye, *N*	43	42	0	60	54	0
Snellen CDVA, mean	20/32	±20/64	0	20/33	±20/68	1
logMAR CDVA, mean	0.3	±0.4	0	0.3	±0.3	1
Kmax (D), mean	59.5	±8.8	0	57.4	±7.7	0
Positive family history, *N*	8	10	2	7	8	8
Atopic constitution, *N*	34	43	2	55	61	6
Smokers, *N*	11	14	3	19	21	9

*N* Number of patients, *SD* standard deviation, *CDVA* corrected distance visual acuity, *D* diopters, *logMAR* logarithm of the minimal angle of resolution, *Kmax* maximum keratometry

At the 1-year post-CXL follow-up, progression had halted in 94 of the 102 eyes that were still in the study (92 %). The remaining eight eyes had progressed, with a mean increase in Kmax of 3.9 D had progressed, with a mean increase in Kmax of 3.9 D (range 1.40–9.40 D). Both visual acuity and Kmax had improved significantly 1 year after CXL treatment. On average, LogMAR CDVA improved from 0.30 to 0.21 (*P* < 0.01), and Kmax decreased from 57.2 to 56.2 D (*P* < 0.01).

### Univariable analysis

The outcomes of our univariable analysis of the initial treatment cohort and the current validation cohort are summarized in Table 2. Both age (β coefficient: 0.006, *P* = 0.04) and pretreatment CDVA (β coefficient: −0.385, *P* < 0.01) were associated with a change in visual acuity. Of those two factors, only pretreatment CDVA had been identified in the initial cohort.

**Table 2:** Univariable analysis of baseline characteristics related to treatment effect in keratoconus patients in the initial and validation cohorts 1 year after corneal crosslinking treatment

	Difference in CDVA (logMAR)					
	Initial cohort			Validation cohort		
	β coefficient^§^	95 % CI	*P* value	β coefficient^§^	95 % CI	*P* value
Age (years)	0.001	−0.006 to 0.008	0.77	0.006	0.000 to 0.013	0.04^‡^
Male gender	0.041	−0.079 to 0.162	0.50	0.066	−0.040 to 0.172	0.22
Positive family history	0.003	−0.168 to 0.173	0.97	−0.061	−0.288 to 0.166	0.60
Atopic constitution	0.121	0.010 to 0.232	0.03^‡^	−0.008	−0.124 to 0.109	0.90
Smoking	−0.047	−0.203 to 0.109	0.54	0.070	−0.065 to 0.205	0.31
Spherical equivalent (D)	−0.002	−0.018 to 0.013	0.99	−0.003	−0.023 to 0.018	0.80
LogMAR UDVA pretreatment	−0.180	−0.290 to −0.070	<0.01^‡^	−0.011	−0.119 to 0.096	0.83
LogMAR CDVA pretreatment	−0.523	−0.641 to −0.405	<0.01^‡^	−0.385	−0.545 to −0.224	<0.01^‡^
Kmax pretreatment (D)	−0.009	−0.016 to −0.003	<0.01^‡^	0.001	−0.006 to 0.008	0.68
Cone eccentricity (mm)	0.098	0.029 to 0.168	<0.01^‡^	0.026	−0.030 to 0.081	0.36
Central corneal thickness (µm)	0.001	0.000 to 0.003	0.04^‡^	0.000	−0.001 to 0.002	0.80

	Difference in maximum keratometry (Kmax)					
	Initial cohort			Validation cohort		
	β coefficient^§^	95 % CI	*P* value	β coefficient^§^	95 % CI	*P* value
Age (years)	0.044	−0.130 to 0.102	0.13^†^	−0.029	−0.087 to 0.029	0.33
Male gender	1.222	0.272 to 2.172	0.01^‡^	0.404	−0.572 to 1.381	0.41
Positive family history	0.693	−0.689 to 2.075	0.32	0.663	−1.441 to 2.766	0.53
Atopic constitution	0.246	−0.679 to 1.171	0.60	0.142	−0.928 to 1.213	0.79
Smoking	−0.417	−1.688 to 0.854	0.52	0.674	−0.578 to 1.925	0.29
Spherical equivalent (D)	0.104	−0.022 to 0.230	0.12^†^	−0.072	−0.259 to 0.115	0.45
LogMAR UDVA pretreatment	−0.871	−1.791 to 0.049	0.06^†^	0.162	−0.832 to 1.156	0.75
LogMAR CDVA pretreatment	−0.771	−2.062 to 0.520	0.24	0.541	−1.117 to 2.198	0.52
Kmax pretreatment (D)	−0.039	−0.091 to 0.014	0.14^†^	−0.046	−0.109 to 0.017	0.15^†^
Cone eccentricity (mm)	0.957	0.400 to 1.515	<0.01^‡^	0.356	−0.151 to 0.863	0.17^†^
Central corneal thickness (µm)	0.011	0.001 to 0.023	0.04^‡^	0.003	−0.012 to 0.018	0.70

*CDVA* Corrected distance visual acuity, *logMAR* logarithm of minimal angle of resolution, *CI* confidence interval, *D* diopters, *Kmax* maximum keratometry, *UDVA* uncorrected distance visual acuity

^§^ß coefficient is the value referring to how a dependent variable will change, per unit increase in the predictor variable

^‡^ *P* < 0.05 indicates significance and this factor is included in multivariable analysis

^†^ *P* values ≤ 0.20 were also included in the multivariable analysis

None of the baseline factors was significantly associated with Kmax outcome in the validation cohort. Because pretreatment Kmax and cone eccentricity demonstrated a trend toward association (β coefficient: −0.046, *P* = 0.15 and β coefficient: 0.356, *P* = 0.17, respectively), they were entered in the multivariable analysis. In the initial cohort, gender, cone eccentricity, and corneal thickness were associated with Kmax outcome.

### Multivariable analysis

Table 3 summarizes the results of the multivariable analyses in both cohorts. In the validation cohort, age (β coefficient: 0.007, *P* = 0.03) and pretreatment CDVA (β coefficient: −0.476, *P* < 0.01) were related independently to a change in visual acuity at the 1-year follow-up visit. Age was not identified as an independent predictor in the initial cohort. With respect to change in Kmax, cone eccentricity was confirmed as an independent predictor of CXL outcome 1 year after treatment (β coefficient: 0.655, *P* < 0.01).

**Table 3:** Multivariable analysis of predictors of treatment effect in keratoconus patients in the initial and validation cohort 1 year after corneal crosslinking treatment

	Difference in CDVA (logMAR)					
	Initial cohort			Validation cohort		
	β coefficient^§^	95 % CI	*P* value	β coefficient^§^	95 % CI	*P* value
Age (years)	−0.002	−0.008 to 0.004	0.50	0.007	0.001 to 0.013	0.03^‡^
Atopic constitution	−0.048	−0.129 to 0.023	0.18			
logMAR UDVA	0.069	−0.083 to 0.221	0.37			
logMAR CDVA	−0.628	−0.997 to −0.258	<0.01^‡^	−0.476	−0.680 to −0.271	<0.01^‡^
Kmax (D)	0.005	−0.001 to 0.011	0.13			
Cone eccentricity (mm)	0.026	−0.044 to 0.096	0.46			
Central corneal thickness (µm)	0.000	−0.001 to 0.001	0.53			

	Difference in maximum keratometry (Kmax)					
	Initial cohort			Validation cohort		
	β coefficient^§^	95 % CI	*P* value	β coefficient^§^	95 % CI	*P* value
Male gender	−0.823	−1.923 to 0.277	0.14			
Spherical equivalent (D)	0.103	−0.045 to 0.251	0.17			
logMAR UDVA	−0.017	−1.105 to 1.071	0.98			
Kmax (D)	−0.009	−0.068 to 0.050	0.77	0.012	−0.059 to 0.083	0.74
Cone eccentricity (mm)	0.709	0.117 to 1.301	0.02^‡^	0.655	0.210 to 1.101	<0.01^‡^
Central corneal thickness (µm)	0.001	−0.010 to 0.012	0.84			

*CDVA* Corrected distance visual acuity, *logMAR* logarithm of minimal angle of resolution, *CI* confidence interval, *D* diopter, *Kmax* maximum keratometry, *UDVA* uncorrected distance visual acuity

^§^ß coefficient is a value referring to how a dependent variable will change, per unit increase in the predictor variable

^‡^ *P* < 0.05 indicates significant values

Visual acuity and cone eccentricity were found to be the sole repeatable and independent factors influencing outcomes of keratoconus patients undergoing CXL, demonstrating that patients with lower pretreatment visual acuity are more likely to benefit from CXL (in terms of visual acuity), and patients with more central cones will benefit more in terms of cone flattening.

### Validation of prediction model

The following equation was used in the initial model to predict the change in logMAR CDVA 1 year after CXL [23]:

$${\text{Difference in logMAR CDVA one year after CXL}} = ( - 0.518 \; \times \; {\text{baseline logMAR CDVA}}) \; +\,0.043.$$

This model showed robust predictive value in the initial treatment cohort (*R* ^2^ = 0.45), explaining 45 % of the variation in CDVA. Validation of the model, in which the validation cohort data are entered into the existing prediction model, showed a mediocre fit (*R* ^2^ = 0.18), only explaining 18 % of the variation. It was not possible to create a better prediction model based on the validation dataset (*R* ^2^ = 0.19). With respect to change in Kmax 1 year after CXL, the model showed limited predictive value in our initial cohort (*R* ^2^ = 0.15) [23]. Fitting a new model to the validation data showed even worst predictive value (*R* ^2^ = 0.02).

Although postoperative CDVA was accurately predicted based on pretreatment patient characteristics in the original study, both visual acuity and maximum keratometry were not predictable for individual patients in this validation study.

## Discussion

The aim of this study was to validate and test the reproducibility of previously determined predictors of CXL effectiveness in a new treatment cohort. This validation cohort confirmed that pretreatment visual acuity and cone eccentricity are the only two independent factors for predicting change in postoperative CDVA and Kmax, respectively. Repeatability of those results is essential to apply these findings in practice and to guide clinicians in their decision-making process.

With respect to cone flattening and visual acuity development, the clinical outcomes following CXL in our cohorts are consistent with previous studies [11, 27, 28]. This again underscores the ability to compare our results to other populations that were treated using the Dresden protocol. Our initial and validation cohorts are relatively large, and only a limited number of cases were lost to follow-up. Interestingly, the univariable analysis revealed major differences between the initial and validation datasets, demonstrating the variability among outcomes when inter-factor correlation is not taken into account. Some baseline measurements are interrelated and therefore could potentially be (incorrectly) identified as predictors when correlated to a true predictor. The predictive factors derived from our multivariable analysis were consistent between the study cohorts, which reflects good reproducibility and stresses the importance of performing a multivariable analysis. The clinical implication of these results is that patients with lower pretreatment visual acuity are more likely to benefit from CXL (with respect to visual acuity), and patients with more central cones will benefit more in terms of cone flattening. This is consistent with other studies in which patients with a pretreatment CDVA of 20/40 or worse had significant visual improvement [16, 17]. Therefore, it might be advisable to explain to patients with low pretreatment CDCVA that improvement might be expected, while on the other hand, this is not likely for patients with pre-existent high CDVA.

Our finding that Kmax is more likely to flatten in eyes with a more central cone is in concordance with results from Greenstein et al. [20]. This latter finding might be due to exposure to UV light perpendicular to the center of the cornea during CXL. The peripheral cornea receives light rays that are less potent due to their oblique incidence. The UV light source used in this study is in accordance with this principle. Furthermore, it is known that CXL and aberrations interact and it could be that aberrations in the vicinity of a peripheral cone alter the angle of incidence of the light rays on the corneal surface even further, causing deflection and thereby resulting in less UV light penetration in the cornea and lesser treatment results [29]. Therefore, a more central cone is likely to be treated more effectively, resulting in more flattening of the cone. Focusing the UV light on the cone instead of the center of the cornea could be considered for treatment customization in patients with more peripheral cones.

Our initial study cohort did not identify age as a predictive factor for either treatment outcomes. However, in the validation dataset, younger patients benefitted significantly more with respect to visual acuity. Soeters et al. also identified age as a prognostic factor; interestingly, they also found that their younger patients had more centrally located cones [14]. Léoni-Mesplié et al. reported that disease progression in pediatric patients is more aggressive than in adults, suggesting that CXL treatment is more effective at preventing deterioration in pediatric patients [30].

Consistent with our initial study, a variety of factors associated with keratoconus were not identified as independent contributors to the effectiveness of CXL treatment. These factors include gender, family history, atopic constitution, smoking history, spherical equivalent, logMAR UDVA, Kmax, and central corneal thickness; none of these factors were predictive in terms of changes in visual acuity or Kmax 1 year after CXL. However, using univariable analyses, other studies found that pretreatment corneal thickness ≤450 µm and Kmax ≥54 D were predictors of a decrease in Kmax [16, 17, 19]. Although we also found a relationship between corneal thickness and Kmax flattening in our initial univariable analysis, this relationship did not hold when examined in a multivariable analysis. This finding was not replicated in the univariable analysis of our validation cohort. Previously, Greenstein et al. used a multivariable approach and found that patients with higher keratometry readings showed more improvement in response to CXL [16]. However, this finding is not supported by our data. Additional analysis using a dichotomous cut-off of 54 D was not associated with either outcome parameter (data not shown). One explanation for this difference in findings could be the difference in sample size and the fact that Greenstein et al. examined a heterogeneous study cohort that included both patients with keratoconus and patients with post-LASIK ectasia.

Creating a model for the prediction of individual Kmax after CXL was ultimately cumbersome and yielded little additional clinical value. Moreover, our initial reliable model for the prediction of CDVA could not be confirmed in this validation study, which is an additional argument why validation studies are extremely important. The inability to validate this prediction model can be due to either overfitting of the original model or to large individual variation in the reaction to CXL treatment. Both options leading to the conclusion that the formerly proposed prediction model for individual visual outcomes after CXL treatment should not be applied for patient counseling.

In conclusion, the clinical implication of these results is that patients with lower pretreatment visual acuity are more likely to benefit from CXL (with respect to visual acuity), and patients with more central cones will benefit more in terms of cone flattening. Repeatability of those predictors supports applicability for the decision-making process of clinicians. Furthermore, those results can be used to guide customization of the crosslinking treatment.
